# Direct Analysis in Real Time by Mass Spectrometric Technique for Determining the Variation in Metabolite Profiles of *Cinnamomum tamala* Nees and Eberm Genotypes

**DOI:** 10.1100/2012/549265

**Published:** 2012-06-04

**Authors:** Vineeta Singh, Atul Kumar Gupta, S. P. Singh, Anil Kumar

**Affiliations:** ^1^Department of Molecular Biology & Genetic Engineering, College of Basic Sciences and Humanities, G. B. Pant University of Agriculture and Technology, Uttarakhand, Pantnagar 263145, India; ^2^Department of Pharmacology & Toxicology, College of Veterinary Science, G. B. Pant University of Agriculture and Technology, Uttarakhand, Pantnagar 263145, India

## Abstract

*Cinnamomum tamala* Nees & Eberm. is an important traditional medicinal plant, mentioned in various ancient literatures such as Ayurveda. Several of its medicinal properties have recently been proved. To characterize diversity in terms of metabolite profiles of *Cinnamomum tamala* Nees and Eberm genotypes, a newly emerging mass spectral ionization technique direct time in real time (DART) is very helpful. The DART ion source has been used to analyze an extremely wide range of phytochemicals present in leaves of *Cinnamomum tamala*. Ten genotypes were assessed for the presence of different phytochemicals. Phytochemical analysis showed the presence of mainly terpenes and phenols. These constituents vary in the different genotypes of *Cinnamomum tamala*. Principal component analysis has also been employed to analyze the DART data of these *Cinnamomum* genotypes. The result shows that the genotype of *Cinnamomum tamala* could be differentiated using DART MS data. The active components present in *Cinnamomum tamala* may be contributing significantly to high amount of antioxidant property of leaves and, in turn, conditional effects for diabetic patients.

## 1. Introduction

The genus *Cinnamomum* belonging to the family Lauraceae comprises 270 species which occur naturally in Asia and Australia. About 20 species occur in India. The etymology is derived from the Greek word “Kinnamomon” (meaning spice). The Greeks borrowed the word from Phoenicians, indicating that they traded with the East from early times. The specific epithet “tamala” is after a local name of the plant in India. *Cinnamomum tamala* Nees and Eberm. is an evergreen, medium-sized tree (attaining 8–12 meters height and a girth of 150 cm), found in India along the North-Western Himalayas, in Sikkim, Assam, Mizoram, and Meghalya [1]. It is also found in tropical and subtropical Asia, Australia, Pacific region, and South Asia [2, 3]. It is distributed from near Indus to Bhutan [4]. Natural stands of *C. tamala* are mostly found in shady moist habitats. The leaves, known as tejpat, tejpatta, or tejpata in Hindi, tamalpatra in Marathi, and Indian Cassia in English, are usually olive green in color, may have some brownish spots and have three veins down the length of the leaf. The leaves of* Cinnamomum tamala *are used extensively in the cuisines of India (particularly in the Moghul cuisine of North India) and as spice in the food industry because of its special aroma [5], that is, clove-like-taste and pepper like odour. It also acts as an insect repellent. In Kashmir they are used as a substitute for paan (betel leaves). It is also used in industries as fragrance component in soaps, detergents, cosmetics and perfumes, and toothpastes. The leaves of this tree have medicinal properties and are used in treatment of numerous ailments [6, 7]. It is used as food, fodder, medicine, and timber in Uttarakhand Himalayan region [8].

Ayurveda describes the use of leaves of tejpatta in the treatment of ailments such as anorexia, bladder disorders, dryness of mouth, coryza, diarrhoea, nausea, and spermatohoea [7]. The leaf of *C. tamala* is a brain tonic, antihelminthic, diuretic, is good for the liver and spleen and is useful in inflammation. Its bark is useful for the treatment of gonorrhoea [9]. Besides these, various pharmacological activities have been detected in natural products from *Cinnamomum *species. The essential oil from *Cinnamomum tamala* exhibits antifungal [10, 11], antibacterial [12], antidermatophytic [13], antihypercholesterolaemic, and antihyperglycaemic effects [14]. It is also used medicinally as a carminative, an anti flatulent, a diuretic, and in the treatment of cardiac disorders [3], analgesic in dental preparations [15–17] due to presence of eugenol (4-hydroxy-3-methoxy allylbenzene). Different extracts from leaves of *C. tamala *have shown antiinflammatory [18], antioxidant [19], antiulcer [20], anticarcinogenic [21], antidiarrhoeal [22] effects, antidiabetic [23, 24] which is mainly contributed by Cinnamaldehyde (3-phenyl-2- propenal), a potential antidiabetic agent [25].

Essential oil extracted from the leaves contains majorly polyphenols, monoterpenoids, and sesquiterpenoids including phellandrene, eugenol, linalool, and some traces of *α*-pinene, p-cymene, *β*-pinene and phenylpropanoids [26], camphene, cinnamyl acetate, camphor, *β*-caryophyllene, [27, 28], myrcene, limonene [29, 30]. Two chemotypes of *C. tamala* were reported, namely, cinnamaldehyde type and eugenol type [31]. Leaves contain essential oil (Eugenol and Isoeugenol), and bark contains 70–80% cinnamic aldehyde.

Direct Analysis in Real Time (DART) is an atmospheric pressure that instantaneously ionizes gases, liquids and solids in open air under ambient conditions. Direct Analysis in Real Time (DART) has been coupled to the AccuTOF atmospheric pressure ionization mass spectrometer to permit high-resolution, exact mass measurements of gases, liquids, and solids [32]. DART is based on the atmospheric pressure interactions of long-lived electronic excited-state atoms or vibronic excited-state molecules with the sample and atmospheric gases. It was among the first ambient ionization techniques not requiring any sample preparation, as ionization can take place directly on the sample surface so plant products can be analyzed directly without any sample preparation [33–35]. In view of the ease with which natural products can be analyzed by DART MS, it was thought that DART MS could be a suitable tool for the chemical profiling of the different landraces of *Cinnamomum tamala* leaves. The presence of phytochemicals can easily be assessed so that the leaves can further be scientifically validated and can be used for combating against the dreadful disease like diabetes.

## 2. Material and Methods

### 2.1. Collection of Sample

**Figure 1:**
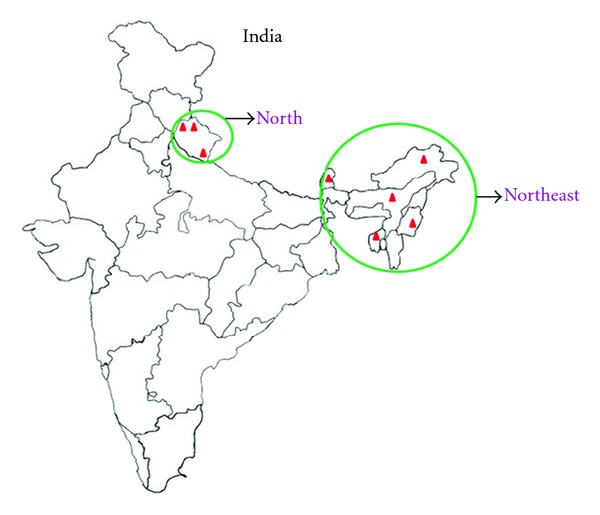
India's map representing the area (Northeast and North) marked by green circle from where the *Cinnamomum* genotypes were procured.

Seven cultivars were procured from IISR Calicut, namely, A11, A12, B12, C1, C2, C3, D11, all from northeast, and three were collected from north Uttarakhand, namely, UM, UC, UH as indicated in Figure 1. The leaf samples were of mature stage and were collected in the month of Feburary.

### 2.2. Instrumentation

 The mass spectrometer used was a JMS 100 TLC (Accutof) atmospheric pressure ionization time of flight mass spectrometer (Jeol, Tokyo, Japan) fitted with a DART ion source. The mass spectrometer was operated in positive ion mode with resolving power of 6000 (full width at half maximum). The orifice 1 potential was set to 15 V, resulting in minimal fragmentation. The ring lens and orifice 2 potential were set to 13 and 5 V, respectively. Orifice 1 was set to a temperature of 100°C. The RF ion guide potential was 300 V. The DART ion source was operated with helium gas flowing at approximately 4.0 L/min. The gas heater was set to 300°C. The potential on the discharge needle electrode of the DART source was set to 3000 V, electrode 1 was 100 V and the grid was at 250 V. Freshly cut pieces of *C. tamala* were positioned in the gap between the DART source and mass spectrometer for measurements. Data acquisition was from m/z 50.0 to 1000.0. Exact mass calibration was accomplished by including a mass spectrum of neat polyethylene (PEG) glycol (1 : 1 mixture PEG 200 and PEG 600) in the data file. m-Nitrobenzyl alcohol was also used for calibration. The mass calibration was accurate to within 0.002 u. Using the Mass Center software, the elemental composition could be determined on selected peaks.

### 2.3. Statistical Analysis

Triplicate samples of each land race were subjected for DART analysis, and results were reproducible. PCA analysis was carried out using JMP 8.0 statistical analysis software.

## 3. Result and Discussion

*Cinnamomum tamala *leaves mainly consist of a number of polyphenols and terpenoids. The compounds reported in plants are listed in Table 1.

**Figure 2:**
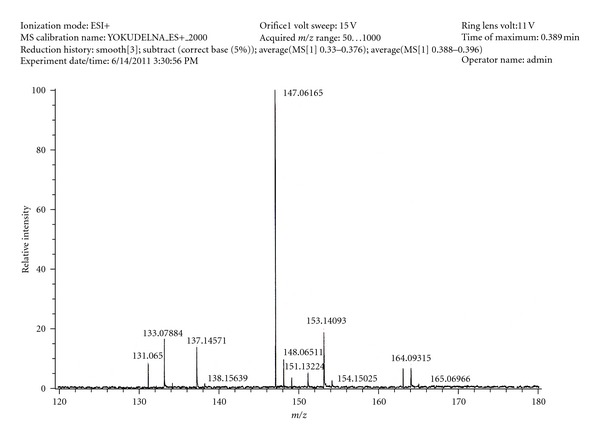
DART MS *C. tamala* leaf of the genotype A11.

The presence of these compounds in different genotypes procured from IISR Calicut (A11, A12, B12, C1, C2, C3, D11) and collected from Uttarakhand (UM, UC, UH) was analysed successfully through DART MS. A representative DART MS of *Cinnamomum tamala* is given in Figure 2.

The DART MS showed the presence of peak m/z 164 which correspond to the major constituent eugenol in leaves of all the ten genotypes. The result was corresponded with another study in which chemotype of *Cinnamomum tamala* leaf oil rich in eugenol was reported from Northeast India [36]. Isoforms of eugenol, that is, methyl eugenol and eugenol acetate showed peak at m/z 178 and 206, respectively. The peak of m/z 132, the peak of cinnamaldehyde, was observed in A11, B12, D11, UH, and UC. This was in contrast to study of Bradu and Sobti [37] who reported cinnamaldehyde as major constituents of *Cinnamomum tamala* leaf oil. Some of the derivatives of cinnamaldehyde were present showing peak at m/z 148 and 176. Presence of certain naturally occurring monoterpenoids and sesquiterpenoids in these genotypes was also observed. Peak m/z at 154 which could be attributed due to linalool was observed only in A12, B12, and D11. Besides this peak of other terpenes was observed at m/z, 126, 166, 180 corresponding to 6-Methyl-5-heptene-2 one, ethyl vanillin, coniferyl alcohol, respectively. Since some of the terpenes have same molecular weight, it was not possible to distinguish them from DART MS alone. The peak at m/z 150 could be due to carvone, carvacrol, or p-cymene-8-ol, and peak at m/z 136 could be of *α*-pinene, camphene, myrcene, limonene, phellandrene. Since they have the same molecular formula, a distinction could not be made. These differences in the spectra of the different cultivars are shown in Table 2.

**Figure 3:**
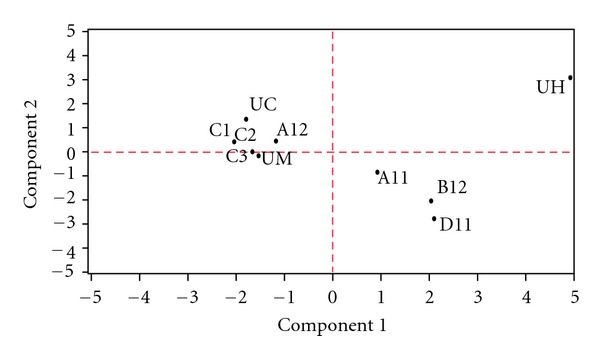
PCA score plot of the abundances of the various ions in the DART mass spectra of the leaves of various *Cinnamomum *genotypes.

It is not easy to identify and differentiate all the components independently based on their mass spectral data. MS base chemical profiling generates complex data sets which need sophisticated software to enable interpretation. Visualization is a key aspect as the data contained a number of variables. PCA is an indirect ordination technique for obtaining a low-dimensional representation of multivariate data so that the data can be explored visually in a two-dimensional PCA correlation biplot and any structure in the data identified. PCA groups the samples solely on information on the measured data and does not need any extra knowledge about the sample and therefore can be used to summarize and visualize the structure of the data. The mass spectral data for all the ten *cinnamomum* leaves were subjected to PCA using 12 variables (abundances at m/z 132, 134, 136, 148, 150, 152, 154, 164, 176, 178, 206, 220). The PCA score plot clearly brings out the relationship among all the* cinnamomum* data, and all the ten sets are clearly separated (Figure 3). It seems that genotype UH is completely different from all other genotypes whereas A11, B12, and D11 are found to have similarity. Rest six genotypes (C1, C2, C3, UM, A12, and UC) are much closer to each other. It is evident from the study that PCA effectively served the purpose and all the *cinnamomum* genotypes could be differentiated by this method.

## 4. Conclusion 

 The combination of ion source with a high-resolution time-of-flight mass spectrometer permits rapid qualitative and quantitative analysis of a wide variety of materials. The DART MS of the leaves of *cinnamomum tamala* could be recorded without any kind of sample preparation. The abundances of the characteristics phenols and terpenes were different in all the ten genotypes. Although peak corresponding to cinnamaldehyde was observed in some of the genotypes, the presence of eugenol was assessed in almost all the genotypes. This compound may be responsible for highest amount of antioxidant activity of the plant which may be enhanced due to the synergestic effect of different kind of terpenes present. Therefore the plant can be used to combat the diseases including diabetes caused by different oxidative stress. PCA showed the expected grouping of genotypes. 

## Figures and Tables

**Table 1:** Exact mass data from the DART mass spectra of *Cinnamomum tamala. *

Molecular weight	Measured mass	Calculated mass	Molecular formula	Remarks
132	131.06500	132.15922	C_9_H_8_O	Cinnamaldehyde
134	133.07884	134.21816	C_10_H_14_	p-cymene
136	135.14386	136.23404	C_10_ H_16_	*α*-pinene, Camphene, Myrcene, limonene, phellandrene
148	148.06511	148.15862	C_9_H_8_O_2_	Cinnamic acid
150	150.13288	150.21756	C_10_H_14_O	Carvone, p-cymene-8-ol
152	151.13224	152.23344	C_10_H_16_O	camphor
154	154.15025	154.24932	C_10_H_18_O	Linalool,1,8 cineole
164	164.09315	164.20108	C_10_H_12_O_2_	Eugenol
176	175.09068	176.21178	C_11_H_12_O_2_	Cinnamyl acetate
178	177.13526	178.22766	C_11_H_14_O_2_	Methyl eugenol
206	205.22984	206.23776	C_12_H_14_O_3_	Eugenol acetate
220	220.13956	220.35046	C_15_H_24_O	*β*-caryophyllene oxide

**Table 2:** DART mass spectral data of A11 (1), A12 (2), B12 (3), C1 (4), C2 (5), C3 (6), D11 (7), UM (8), UC (9), UH (10) cultivars of *Cinnamomum tamala *leaves.

m/z	*Cinnamomum tamala* genotypes									
	A11	A12	B12	C1	C2	C3	D11	UM	UC	UH
127	−	−	+	−	+	+	−	+	+	+
131	+	−	+	−	−	−	+	−	+	+
132	+	−	+	−	−	−	+	−	+	+
133	+	−	+	−	−	−	+	−	+	+
135	−	−	+	−	−	−	+	−	+	+
137	+	−	+	−	+	−	+	−	+	+
145	−	−	+	+	+	+	+	+	+	+
147	+	−	+	−	−	−	+	−	−	+
148	+	−	+	−	−	−	+	−	−	+
150	+	+	+	*‒*	*‒*	+	+	*‒*	+	+
151	+	−	+	−	−	−	+	−	−	+
153	+	−	+	−	−	−	+	−	−	+
154	+	−	+	−	−	−	+	−	−	+
163	+	+	+	+	+	+	+	+	+	+
164	+	+	+	+	+	+	+	+	+	+
165	+	+	+	+	+	+	+	−	+	+
166	*‒*	+	+	+	+	+	*‒*	*‒*	+	+
175	−	−	+	−	−	−	−	−	−	+
177	−	−	+	−	−	−	−	−	−	+
180	−	+	+	+	+	+	−	+	+	+
193	+	+	+	−	+	+	*‒*	+	+	+
205	−	−	+	−	−	−	−	−	+	−
220	−	−	+	−	−	−	+	−	+	−
287	−	+	+	−	−	−	−	+	−	+
293	+	−	+	−	−	−	+	−	−	+
445	+	+	+	+	+	+	+	+	+	+
447	+	+	+	+	+	+	+	+	+	+
448	+	+	+	+	+	+	+	+	+	+
449	+	+	+	+	+	+	+	+	+	+

+ Present, −Absent.

## Abbreviations

DART: Direct analysis in real time
PCA: Principal component analysis
TOF: Time of flight.
